# Technical and clinical evaluation of a closed loop TIVA system with SEDLine^TM^ spectral density monitoring: Multicentric prospective cohort study

**DOI:** 10.1186/s13741-019-0130-2

**Published:** 2020-01-09

**Authors:** Claudia Castellanos Peñaranda, Fabián D. Casas Arroyave, Francisco J. Gómez, Paola A. Pinzón Corredor, Juan M. Fernández, Marcela Velez Botero, Juan D. Bohórquez Bedoya, Carlos Marulanda Toro

**Affiliations:** 1 Anesthesiology and Resuscitation, Fundación Universitaria Ciencias de la Salud-FUCS, Bogotá, Colombia; 2 Anesthesiology and Resuscitation, Faculty of Medicine, Universidad de Antioquia, Medellín, Colombia; 3 Faculty of Medicine, Universidad de Antioquia, Anesthesiologist, Hospital Universitario San Vicente Fundación, Medellín, Colombia; 4 Faculty of Medicine, Universidad de Antioquia, Anesthesiologist, IPS Universitaria, Ambulatory Headquarters, Medellín, Colombia; 5 Fundación Universitaria Ciencias de la Salud- FUCS, Anesthesiologist, Hospital San José, Bogotá, Colombia; 6 Bioengineer, Faculty of Engineering, Universidad de Antioquia, Medellín, Colombia; 7 Anesthesiologist, IPS Universitaria, Ambulatory Headquarters, Medellín, Colombia

**Keywords:** Closed loop, Total intravenous anesthesia, Propofol, Remifentanil, SEDline, Spectral density

## Abstract

**Introduction:**

Closed loop total intravenous anesthesia is a technique in which the patient’s hemodynamic and anesthetic depth variables are monitored, and based on this information, a computer controls the infusion rate of drugs to keep them within pre-established clinical parameters.

**Objective:**

To describe the technical and clinical performance of a closed loop system for total intravenous anesthesia with propofol and remifentanil, using the SEDLine^TM^ monitor

**Design:**

Multicentric prospective cohort study

**Setting:**

Surgery room

**Patients:**

ASA I-II undergoing elective surgery

**Measurements:**

The authors designed a closed loop system that implements a control algorithm based on anesthetic depth monitoring and the Patient State Index (PSI^TM^) of the SEDLine monitor for propofol, and on hemodynamic variables for remifentanil. The measurement of clinical performance was made based on the percentage of PSI^TM^ maintenance time in the range 20–50. Precision analysis was evaluated by measuring median performance error (MDPE) can be defined as the median difference between actual and desired values, which refers to the degree of precision in which the controller is able to maintain the control variable within the objective set by the anesthesiologist; it represents the direction (over-prediction or under-prediction) of performance error (PE) rather than size of errors, which is represented by MDAPE, median absolute percentage error, Wobble index, which is used for measuring the intrasubject variability in performance error.

**Results:**

Data were obtained from 93 patients in three healthcare centers. The percentage of PSI^TM^ maintenance time in the 20–50 range was 92% (80.7–97.0). MDPE was 10.7 (− 11.0–18.0), MDAPE 21.0 (14.2–26.8) and wobble 10.7 (7.0–16.9). No adverse surgical or anesthetic events were found.

**Conclusions:**

The closed loop total intravenous anesthesia system with SEDLine developed by the authors was used without major complication and appear to be feasible its use in clinical performance.

## Introduction

Total intravenous anesthesia (TIVA) is a technique administered using two methods: open loop or target-controlled infusion system (TCI) through manual intravenous infusion, and closed loop control system. Closed loop is an automated control system based on the feedback principle, which calculates and administers the drug according to the target or effective-site concentrations defined by the anesthesiologist. The open loop system has been shown to be inaccurate for predicting and maintaining the desired clinical effect, since pharmacokinetic and pharmacodynamic mathematical models have little physiological correlation and great interindividual variation (Leslie et al., 2008; Ting et al., 2004; Reboso et al., 2012; Struys et al., 2001), overestimating doses of inducing drugs, with adverse hemodynamic effects. On the other hand, closed loop TIVA has shown better performance (Struys et al., 2001), and is able to handle interindividual variations and the effects of drugs.

In a closed loop system, mean arterial pressure (MAP) and heart rate (HR) are used as controlled variables (Struys et al., 2001) to adjust analgesic infusions (opioids such as remifentanil). However, two variables of greater interest are the control of the depth of hypnosis and monitoring the patient’s level of consciousness, information that allows adjusting propofol infusion. The ideal variable with which to measure and titrate the depth of anaesthesia is not known. However, numerous indices have been developed to measure and titrate the delivery of anaesthetic agents to achieve a targeted depth of anaesthesia. Regarding TIVA closed loop systems, Bispectral Index (BIS^TM^, Covidien Ltd, Ireland) monitoring is commonly used as index of the level of consciousness to guide propofol administration. (Hemmerling & Charabati, 2010) In turn, SedLine^TM^ is a new quantitative index of EEG that evaluates the depth of anesthesia based on a proprietary algorithm, which generates a Patient Status Index (PSI^TM^) between 0 and 100, where PSI^TM^ 100 represents wakefulness and 0, with an isoelectric EEG, suppression. The PSI^TM^ between 20 and 50 is associated with an adequate level of anesthetic depth (Drover & Ortega, 2006). The algorithm analyzes the performance power of specific frequency bands combined with changes in symmetry and synchronization in several cortical regions (Purdon et al., 2015; Rampil, 1998). It uses the L1 and R1 front channels of its sensor and records the alpha activity that best characterizes the anesthetic state.

Both the PSI^TM^ and the BIS^TM^ have proven to be good predictors of loss of consciousness, correlating in all phases of anesthesia (Chen et al., 2002). The PSI^TM^ may be more sensitive and reliable considering that it has a self-normalization technique that depends on interindividual variability in EEG activity and on the response of each brain to different anesthetics. Studies have shown that its use decreases the use of medications such as propofol, improves the profile of early recovery (Drover et al., 2002), is less susceptible to interference by electrocautery, detects periods of electroencephalographic suppression and reports its percentage in the weather (Purdon et al., 2015; Chen et al., 2002; Drover et al., 2002). Index of EEG in anaesthesia can reduce the risk of intraoperative awareness in surgical patients at high risk for awareness compared to using clinical signs the guide to anaesthetic practice (Punjasawadwong et al., 2014).

The authors developed a closed loop system using software based on fuzzy logic, proven in a case report (Gómez Oquendo et al., 2013), a prospective case series (Hemmerling et al., 2013a) and a controlled clinical trial (Casas & Fernandez, 2016), which provided clinically adequate anesthesia and satisfactory operating conditions in all studied patients during the period of automatic control using the BIS^TM^ for anesthetic depth monitoring.

The objective of this study was to describe the technical and clinical performance of the described closed loop TIVA administration system with propofol and remifentanil, in maintaining a target PSI^TM^ with minimal fluctuations, using neuromonitoring with SEDLine^TM^ in adult patients taken to surgery under general anesthesia in three different health Colombian institutions.

## Materials and methods

We conducted a descriptive cohort study, conducted between March 2016 and July 2018 in three healthcare centers: (1) IPS Universitaria, Ambulatory Headquarters (Medellín), (2) Hospital San José (Bogotá D.C.), and (3) S.E.S Hospital de Caldas (Manizales), with a convenience sample of 93 patients. Written informed consent was obtained from each participant before data collection and appropriate measures were taken to minimize risks and maintain confidentiality. The protocol was summited to the Ethical and Research Institutional Board (three universities) and approved by the Bioethics Committees of each institution. The study was nor registered in any research platforms. We followed the STROBE (Strengthening the Reporting of Observational Studies in Epidemiology) guidance in conducting and reporting our investigation.

### Participants

Inclusion criteria were age over 18 years, American Society of Anesthesiologist (ASA) physical status 1–2, planned for undergoing elective noncardiac surgery (general surgery, traumatology and orthopedic surgery, gynecological, urological, plastic, ophthalmology, and otorrinolaryngology surgery) of low or intermediate risk, under general anesthesia by closed loop TIVA technique were studied. Patients with a history of allergy to opioids or any component of propofol, pregnant, with morbid obesity (BMI > 40), who did not consent to their participation and patients requiring analgesic or anesthetic peripheral block prior to surgery were excluded from the study. The participating anesthesiologists were trained in the operation of the equipment. The eligibility criteria were verified and informed consent was completed.

### Procedures and equipment

The authors designed a closed loop system using the PSI^TM^ as a clinical variable for the control of propofol concentration, and HR and BP as control variables for remifentanil infusion.

On the day of surgery at the holding area, a peripheral IV line was established with an 18-gauge IV canula in the forearm and two 3-way keys were placed. In the operating room, patients were monitored with pulse oximetry and non-invasive BP using a ROOT® monitor (Masimo, California) connected to the module and the closed loop software designed by the researchers. Standard monitoring, including 5-lead electrocardiography and capnography were attached and recorded with independent monitors.

The developed control algorithm contains a patented diffuse logic control module that adjusts the dose-response effects in situ of the hypnotic (propofol) and the opioid (remifentanil) agents according to the physiological variables of the patient.

For propofol, the algorithm took into account electromyography parameters, suppression rates, spectral edge frequencies and the PSI^TM^ for dose adjustment. Fuzzy logic was chosen as the best control mechanism, because its linguistic nature allows translating better the decisions of an expert anesthesiologist into equations and curves, in such a way that there are no unexpected answers or very “abrupt” answers, as may be the case with a deterministic control. In other words, for a control system like this, radical scales cannot be presumed if a patient with a PSI value of 51 is awake, but is anesthetized with a value of 50.

Fuzzy logic was also used for remifentanil. Control variables are “changes in hemodynamic variables” and not their absolute values, that is to say, a universal blood pressure value is not taken into account, since a patient can be significantly over-dosed if taken to a MAP of 50 when basal MAP in the anesthetic phase is 60 and naturally resists going below that level. For this reason, the controller analyzes the patient’s sensitivity to induction doses and, during maintenance, modifies the target doses according to new changes in the HR or MAP with respect to the basal “sensitivity” found during induction. Similarly, for an automatic controller, a patient with a MAP of 50 cannot be considered normotensive and is considered hypotensive with MAP at 49.

In case of requiring vasoactive agents or of having alterations in the mentioned variables, the closed loop system allows converting the system in an open one and continuing the TCI infusion in that way. This prevents the system from interpreting a bolus of ethylephrine as a nociceptive response. The system has a button through which the anesthesiologist can anticipate a painful surgical stimulus and apply a bolus of opioid prior to the stimulus without abandoning the closed loop mode, after which the system continues to analyze the hemodynamic changes and can increase even more the target concentration in case of finding disturbances.

**Fig. 1:**
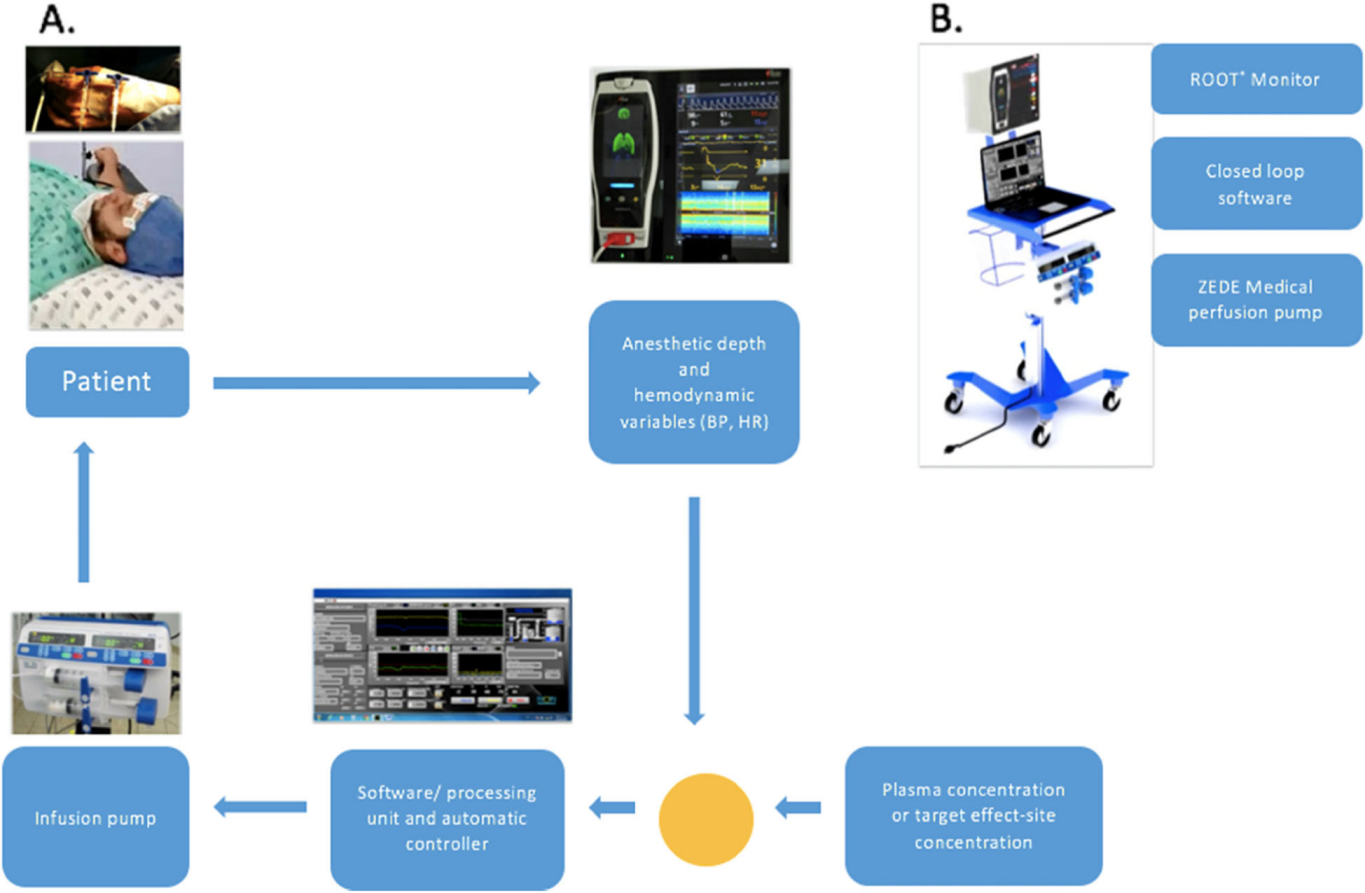
**a** Operation of the total intravenous anesthesia administration system in closed loop. **b** Complete closed loop system module designed by the authors with each of its interconnected components

The EEG sensors for the SEDLine^TM^ monitoring system (Masimo, California), consisting of four channels, are placed in the frontal region (active electrodes L1, L2, R1, and R2, CB ground electrode and CT reference electrode). The PSI^TM^, BP, pulse oximetry, and HR (monitor ROOT®) are connected to the signal processing and control unit, consisting of a computer (Shenzhen Yanzchao Tech for Bioin Soluciones, Intel Ivy Bridge Celeron CPU 1037 U, SODIMM 2GB DDR3 memory) with the algorithm developed by the research team connected to a perfusor (ZEDE Medical, design of Bioin Solutions), with a maximum flow rate of 1200 mL/h and with capacity for two 60-mL syringes, which executes the control orders for the perfusion rates calculated by the closed loop software for propofol and remifentanil. The PSI^TM^ is reported with a delay of 20 seconds while the spectrogram and suppression rates are reported in real time (see Fig. 1)*.*

Once the reception of the signals of the monitor and its recognition by the processing unit is confirmed, the patient is connected to the propofol and remifentanil perfusion equipment. The variables of the model are entered (patient characteristics: age, height, weight, and sex), and the target concentration is selected. The program performs a simulation using the pharmacokinetic parameters according to the Schnider (Absalom et al., 2009) and Minto (Minto et al., 1997) model and, together with the values of the variables, calculates the infusion rate profiles needed to reach the target concentrations defined by the anesthesiologist.

During the surgical procedure, the algorithm adjusts the perfusion rate of propofol every 30 s, re-feeding based on the anesthetic depth reports of the SEDLine^TM^ with the PSI^TM^ value, whose target maintenance is between 20 and 50 (anesthesia 25–50, deep anesthesia 20–25). The closed loop system algorithm has two moments: (a) induction and (b) maintenance, adjusting the rate and duration of propofol delivery differently at each time. During induction, the controller attempts to achieve the target concentration in a phased manner based on the initial calculation—which is in turn based on the pharmacokinetic model (continuously feeding back through the PSI^TM^)—and during maintenance from a target value of the PSI^TM^, but taking into account the existing PSI^TM^ (which is recorded every 10 s).

The perfusion rate of remifentanil is controlled by monitoring the ANALGOSCORE designed and validated by Hemmerling et al. (Hemmerling et al., 2009; Hemmerling et al., 2013b), which assign a score of 3 as adequate analgesic control from the MAP and HR variables. After the surgical procedure, infusions are manually suspended. Once extubated, the patients are transferred to the postanesthetic care unit (PACU) where they are evaluated for 2 h following the surgical procedure.

### Variables and data processing

Anthropometric measurements, sociodemographic, and hemodynamic variables, baseline MAP and HR, and intraoperative data were recorded. For the purposes of this study, the researchers defined hemodynamic instability as a 20% reduction of the baseline sustained systolic BP during two consecutive doses. The variables maintenance time in the PSI^TM^ between 20 and 50 and percentage of technical performance of the controllers were stored by the software, automatically generating a database for each patient. At PACU, anesthesiologists recorded the time of discharge, episodes of postoperative nausea and vomiting (PONV) and the presence or absence of intraoperative recall according to the Michigan classification.

### Statistical analysis

It is a descriptive study, for that reason we don’t report statistical test. Quantitative variables were reported using measures of central tendency and dispersion, after evaluating the criterion of normality through the Kolmogorov-Smirnov test. Qualitative variables were presented as proportions and frequencies. The statistical analysis was performed using SPSS version 20.0 and Excel 2013.

### Outcome variables

The technical performance of the system based on the PSI^TM^ was evaluated using the formulas described by Varvel et al. (Varvel, 1992), which have been widely validated when assessing any device of this nature from a technical point of view. The performance of the systems was evaluated by comparing the predicted and measured values of the concentration in blood or plasma, taking into account that drug concentration in plasma cannot be measured in real time on many occasions, but in others the effect of the evaluated drug, for example, changes in the electroencephalogram processed for hypnotics such as propofol and can be controlled in real time. In these scenarios, performance measures can be calculated in real time. Varvel et al. (Varvel, 1992) recommended 5 indexes to evaluate the performance of these devices:

- Performance error (PE) is the weighted residual of the target value at any given time point; the difference between the actual values and the target value.
- Median performance error (MDPE) is a measure of bias and indicates whether the administration of drugs with the system is systematically above or below the target value.
- Median absolute performance error (MDAPE) indicates the inaccuracy of the TCI system and is a quantitative measure of how far the observed value deviates from the target; its value varies between 20 and 50%.
- Wobble indicates the within-subject variability of the TCI system. In this context, Wobble is a measure of variability in the patient’s PE. The clinically accepted value is 10–20%.
- Divergence is the slope of linear regression between the MDAPE and time. Divergence shows whether inaccuracy of the TCI system changes over time.

All these measures focus on the capacity of the TCI system to achieve and maintain a specific plasma drug concentration, which are appropriate to evaluate the performance of computer-controlled infusion pumps (Hemmerling et al., 2013b).

An ideal TCI system would yield an observation that fits perfectly the predicted value. Therefore, the performance error (MDAPE) and the bias (MDPE) should be close to zero, and the relationship between the observed value and the anticipated value would be unity. The TCI system should also be stable over time, so that oscillation and divergence are as low as possible. PE < 20–40% and MDPE < 10–20% are considered clinically acceptable.

## Results

**Fig. 2:**
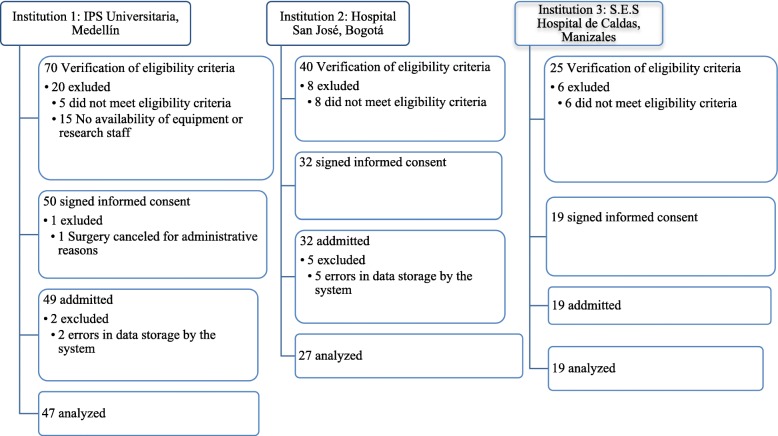
Flowchart of cases registration by institution

One hundred patients were taken to closed loop TIVA; the information of 7 cases was lost due to a storage error in the system. The 93 patients analyzed were distributed as follows: 47 in institution 1, 27 in institution 2, and 19 in institution 3 (Fig. 2).

The average age was 41 years; 52.6% of patients were ASA I, and outpatient procedures were predominant (87.4%). 46.3% of surgeries were plastic surgery, followed by 22.1% of general surgeries (Table 1).

**Table 1:** Basic anthropometric and sociodemographic characteristics of the patients

Care center	Institution 1: IPS Universitaria. Medellín (*n* = 47)	Institution 2: Hospital San José. Bogotá (*n* = 27)	Institution 3: S.E.S Hospital de Caldas (*n* = 19)	Consolidated Global (*n* = 93)
Age (years)*	27 (22–34)	49 (38–68)	48 (38–65)	41 (25–49)
Sex**				
Female	30 (63.3%)	17 (63%)	14 (73.7%)	62 (65.3%)
Male	17 (36.7%)	10 (37%)	5 (26.3%)	33 (34.7%)
Weight (Kg)*	58 (52–65.5)	63 (59–80)	66 (58–83)	60 (54–70)
Height (cm)*	160 (157–167)	162 (158–167)	158 (148–173)	161 (156–168)
BMI (Kg/cm2)*	22 (20.5–24)	25 (22.2–27.7)	27.5 (23.6–29.3)	23.5 (21–26)
Type of surgery **	General surgery 1 (2%) Plastic surgery 32 (67.3%) Otorhinolaryngologic surgery 14 (30.6%)	General surgery 11 (40.7%) Orthopedic surgery 7 (25.9%) Gynecological or urological surgery 9 (33.3%)	General surgery 9 (47.4%) Plastic surgery 2 (10.5%) Otorhinolaryngologic surgery 1 (5.3%) Orthopedic surgery 1 (5.3%) Other 6 (31.6%)	General surgery 21 (22.5%) Plastic surgery 34 (36.5%) Otorhinolaryngologic surgery 15 (16.1%) Orthopedic surgery 8 (8.64%) Gynecological or urological surgery 9 (9.6%) Other 6 (6.4%)
Origin of the patient**				
Outpatient	47 (100)	23 (85.2%)	11 (57.9%)	83 (87.4%)
Inpatient	0 (0)	4 (14.8%)	8 (42.1%)	12 (12.6%)
ASA classification **				
ASA I	38 (79.6%)	5 (18.5%)	6 (31.6%)	50 (52.6%)
ASA II	9 (20.4%)	22 (81.5%)	13 (68.4%)	45 (47.4%)
Basal systolic arterial pressure (mmHg)*	116 (110–125)	130 (116–139)	123 (110–133)	120 (110–130)
Basal mean arterial pressure (map) (mmHg)*	85 (78.5–92.5)	74 (64–84)	86 (83–99)	86 (82–98)
basal diastolic blood pressure (DBP) (mmHg)*	70 (61.5–79.5)	93.6 (83.6–101)	70 (67–75)	70 (64–80)
Basal heart rate (hr) (bpm)***	74 (8.2)	72.1 (10)	79 (12)	74 (8.2)
Surgical time (minutes)***	143 (60.5)	73.8 (33.1)	86 (39.5)	143 (60.5)
Duration of anesthesia (minutes)***	162 (65.1)	84.8 (33.9)	106 (46.9)	74 (9.6)
Analgesic technique**	Opioids 0 (0) Opioids + NSAIDs 30 (63.3) Opioids + NSAIDs + analgesic block 17 (36.7)	Opioids 0 (0) Opioids + NSAIDs 27 (100) Opioids + NSAIDs + analgesic block 0 (0)	Opioids 9 (47.4) Opioids + NSAIDs 0 (0) Opioids + NSAIDs + analgesic block 0 (0) NSAIDs + analgesic block 8 (42.1%) Analgesic block 1 (5.3%) NSAIDs 1 (5.3%)	Opioids 9 (9.67%) Opioids + NSAIDs 57 (61.3%) Opioids + NSAIDs + analgesic block 17 (18.2%) NSAIDs + analgesic block 8 (8.6%) Analgesic block 1 (1.1%) NSAIDs 1 (1.1%)

*Median (interquartile range 25–75) **frequency (percentage %) ***mean (standard deviation)

Clinical performance measurement of the closed loop system was the percentage of maintenance of the PSI^TM^ between 20 and 50, which was 92% (80.7–97.0) (Table 2).

**Table 2:** Clinical outcomes of patients under general anesthesia with closed-loop TIVA technique and SEDLine^TM^ monitoring by institution

Care center	Institution 1: IPS Universitaria. Medellín (*n* = 47)	Institution 2: Hospital San José. Bogotá (*n* = 27)	Institution 3: S.E.S Hospital de Caldas (*n* = 19)	Consolidated Global (*n* = 93)
Percentage of time spent by the PSI^TM^ between 20 and 50*	96 (89–99)	84.8 (70.8–94)	85.6 (78–96.6)	92%
Episodes of hemodynamic instability**	2 (4.1%)	6 (22.2%)	0 (0)	8 (8.4%)
Need for vasopressors**	1 (2%)	6 (22.2%)	0 (0)	7 (7.4%)
Episodes of intraoperative movement**	9 (18.4%)	2 (7.4%)	0 (0)	11 (11.6%)
Surgical complications**	0 (0)	0 (0)	0 (0)	0 (0)
Adverse events**	0 (0)	0(0)	0 (0)	2 (2.1%)
Extubation time (minutes)*	8 (5.5–13)	11 (9–13)	6 (5–8)	8 (6–13)
Need for open-loop change**	3 (6.1%)	0 (0%)	0 (0)	4 (4.2%)
Need for change to halogenated anesthetics**	3 (6.1%)	1 (3.7%)	0 (0)	4 (4.2%)
PACU discharge time (minutes)*	80 (60–100)	100 (44–926)	60 (45–120)	80 (60–100)
Episodes of postoperative nausea and vomiting	0 (0)	0 (0)	0 (0)	0 (0)
Recall of intraoperative events**	1(2.1%)	0 (0)	0 (0)	1(1.1%)
Amount of propofol (mcg/kg/min)*	5.84 (4.6–6.2)	6.05 (5.4–7.3)	5-48 (4.3–5.9)	5.84 (4.6–6.2)
Amount of remifentanil (mcg/Kg/min)*	9.7 (5.5–10.8)	11.78 (8.6–12.8)	8.4 (4.1–9.3)	9.7 (5.5–10.8)

*Non-normal quantitative variables. Median data (interquartile range 25–75) **Qualitative variables. Frequency data (percentage %). *NA* Data not available

**Fig. 3:**
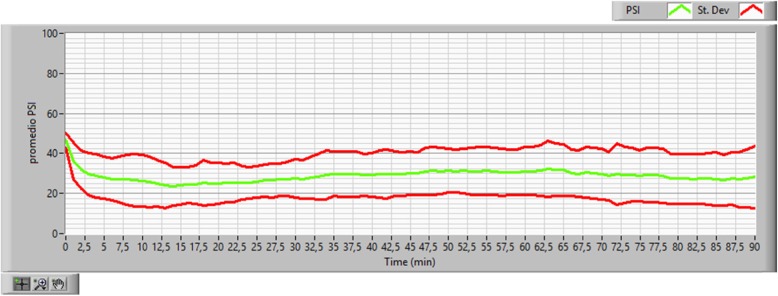
Performance of the PSI^TM^ during the surgical time of all patients. The green line represents the mean of the PSI^TM^, and the red lines the standard deviation for each minute of the surgical procedure

**Fig. 4:**
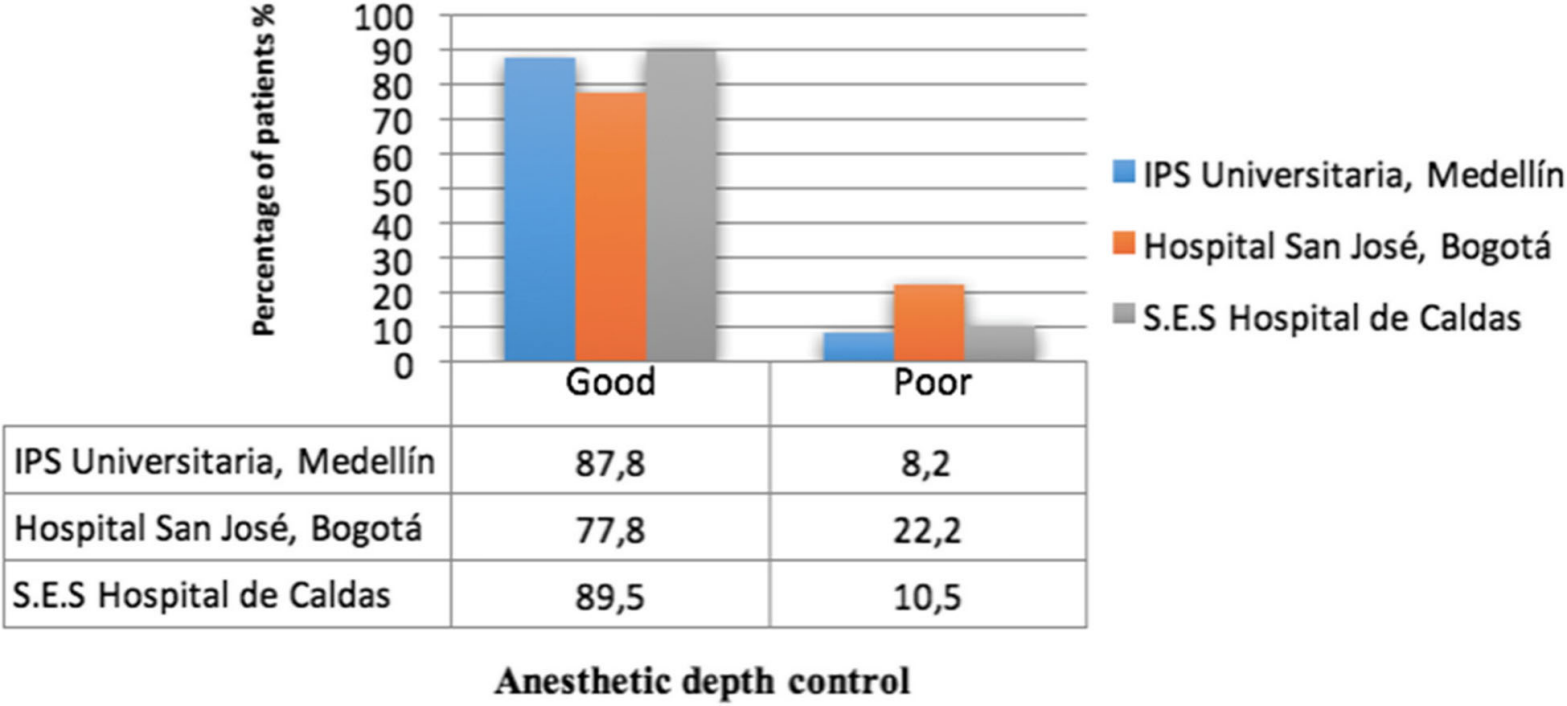
Closed loop control of anesthetic depth in each institution. Control is considered good when the PSI^TM^ is between 20 and 50 in > 70% of anesthetic duration. Control is considered poor when the PSI^TM^ between 20 and 50 is < 70% of anesthetic duration. The length of the bars represents the percentage of patients at each control level

Figure 3 describes the behavior of the PSI^TM^ during the surgical time. At the beginning of the procedure and up to around minutes 7.5 to 10, the PSI levels were high and correlated adequately with the induction phase. In minutes 10 and 90, the PSI^TM^ value stabilized within the desired 20–50 target, which is related to the maintenance phase of direct control by the closed loop. In our study, 85.3% of patients were at this level (Fig. 4).

8.4% of patients had episodes of hemodynamic instability, with vasopressor management in 7.4%. Intraoperative movement episodes were documented in 11.6%. The change of anesthetic technique to open loop and/or use of halogenated ethers occurred in 4.2% of cases. There were no adverse surgical or anesthetic events; there were no episodes of PONV in the PACU. One patient had class 1 intraoperative recall according to the Michigan Scale (isolated auditory perception) related to disconnection of the venous line and consequently of the remifentanil and propofol infusions (Table 2)*.*

The technical performance of the closed loop measured with the MDPE was 10.7 (− 11.0–18.0), with MDAPE 21.0 (14.2–26.8), and Wobble 10.7 (7.0–16.9) (Table 3)*.*

**Table 3:** Technical performance of patients taken to general anesthesia with closed loop TIVA technique and SEDLine monitoring

Care center	Institution 1: IPS Universitaria, Medellín (*n* = 47)	Institution 2: Hospital San José, Bogotá (*n* = 27)	Institution 3: S.E.S Hospital de Caldas (*n* = 19)	Consolidated Global (*n* = 93)
MDPE*	11 (− 18–18)	10.7 (− 10.7–21.4)	10.7 (− 7.1–25)	10.7 (− 11–18)
MDAPE*	21 (14–25)	21.4 (4.2–32.1)	17.6 (14.2-28.5)	21 (14–26.8)
WOBBLE*	7 (7–11)	14.2 (10.7–17.8)	10.7 (7.1-14.2)	10.7 (7–16.9)

MDPE, Technical median performance error

MDAPE, median absolute performance error

WOBBLE, within-subject variability in the infusion system. Median (interquartile range 25–75)

## Discussion

The performance of this automated system for the induction and maintenance of TIVA with propofol and remifentanil, using neuromonitoring with SEDLine^TM^, measured with the percentage of time of maintenance of the PSI^TM^ between 20 and 50, was good in all institutions, being > 70% of the anesthetic duration in 85.3% of patients, value considered adequate as reported for this type of device (Hemmerling et al., 2013a). In the previous works of the authors using the BIS^TM^ for neuromonitoring, the maintenance time between 40 and 60 for the closed loop group was 75.24% (SD 15.78) (Punjasawadwong et al., 2014; Hemmerling et al., 2013a). In our study, maintenance time using the PSI^TM^ between 20 and 50 was 92%. Puri G.D.et al. in a multicenter clinical trial with 121 anesthetized patients with closed loop propofol and fentanyl and neuromonitoring with BIS^TM^, described a percentage of maintenance time in target BIS^TM^ of 81.4% (76-89) (Puri et al., 2016). Hemmerling T.M. et al. in their clinical trial with 93 patients using the McSleepy closed-loop system, had a percentage of maintenance time for the BIS^TM^ of 81% (Punjasawadwong et al., 2014), evidencing that this system has a similar clinical performance and discretely greater than other closed loop systems using the BIS^TM^.

The difference in maintenance in the PSI^TM^ among institutions could be explained by the heterogeneity of the population, the diversity of the surgical procedures, the greater number of ASA II patients and an older age, where the variation in susceptibility to anesthetics is known, being higher in the elderly population (Sepúlveda Voullieme & Abadía, 2013). The difference in the commercial origin of the drugs could cause greater requirements to reach an adequate PSI^TM^ during induction and maintenance phase, generating a confusion factor that the control algorithm cannot include in its analysis.

Most arterial hypotension events were observed during the induction phase, a weakness that contrasts with observations during the same open-loop phase that is related to the generalized intention of anesthesiologists of over dosing patients to ensure adequate anesthetic induction, demonstrating that the presence of an anesthesiologist minimizes the impact on this type of variables in any of the two modalities.

Intraoperative movement in the absence of neuromuscular relaxation is a control variable of intraoperative analgesia. Depending on the type of surgery and in those in which the painful stimulus is greater or has large fluctuations in the operative period, they are more susceptible to presenting poor analgesic control. After the inclusion of the command in the software that allows the anesthesiologist to anticipate the painful stimulus according to the specific moment of the surgery, a significant reduction in episodes of intraoperative movement was identified in the following cases.

This demonstrates that although the closed loop system allows making appropriate adjustments in terms of PK/PD individually by giving anesthesia closer to the hypnosis and nociception targets, the anesthesiologist can never be excluded from the anesthetic act because his knowledge on the development of the surgical procedure and his anticipation skills, analysis of the clinical situation in the operating room and changes in the induction phase are required, thus leaving more repetitive tasks to the closed loop (Scher et al., 2016).

Change to open-loop technique was found in 4 cases: loss of the EEG monitor signal due to sensor failures; loss of the connection between the computer and the PSI^TM^ monitor; inadequate programming of the patient's anthropometric parameters in the computer, leading the system to an inadequate calculation of the infusion rates; and a case in an older adult that required high doses of anesthetic to obtain an adequate PSI^TM^, with episodes of hypotension. These situations did not allow reusing SedLine^TM^ monitors, which can be used for 24 hours maximum as recommended by the manufacturer (Corporation, 2017). The software included limits on the entry of anthropometric data, preventing progress until the typing errors are corrected. In two cases, disconnection of the venous line occurred during the intraoperative period, one of them correlated with the episode of intraoperative recall.

TIVA safety is one of the cornerstones of the proper performance of this technique, confirming the need for the presence and permanent care of an anesthesiologist. The National Patient Safety Agency of the National Health Service of the United Kingdom reported 89 incidents between 2008-2009 related to venous access; there were 5 cases of intraoperative recall (Craft, 2015). Safety and management recommendations for venous lines in TIVA include sound alarms from infusion pumps to detect pressure changes, active surveillance of venous lines, and use of antireflux valves (Barvais et al., 2013).

Time of extubation, considered as the time elapsed between the suspension of the infusions of propofol and remifentanil and the extubation of the patient, was 8 (Mcgee et al., 2006; Gómez Oquendo et al., 2013; Hemmerling & Charabati, 2010; Purdon et al., 2015; Drover & Ortega, 2006; Rampil, 1998; Chen et al., 2002; Drover et al., 2002) minutes for the institutions. These results correlate well with the time to awakening for the closed loop with BIS^TM^ reported by G.D. Puriet et al, which was 8 min (Gómez Oquendo et al., 2013; Hemmerling & Charabati, 2010; Purdon et al., 2015; Drover & Ortega, 2006; Rampil, 1998), and Hemmerling et al. who described a time of 10.1 min (SD 4.7) (Punjasawadwong et al., 2014), Reboso et al. of 9 min (SD 5.0) (Reboso et al., 2012), and in the case series of the authors using BIS^TM^ 9.8 min (SD 4.2) (Casas et al., 2015).

There were no episodes of PONV, which is consistent with several studies that have demonstrated the antiemetic properties of propofol, recommending the use of the TIVA technique in patients considered to be at high risk of PONV (Darnobid, 2015; Gupta et al., 2004; Vasileiou et al., 2009; Gan et al., 2014).

Regarding technical performance, the results show that the system is within the internationally accepted parameters for this type of devices. The publications that evaluate them consider a MDAPE between 20 and 50% as acceptable (Pasin et al., 2017); in this study, it was 21%. The MDPE, whose clinically accepted value is 10–20% (Gan et al., 2014), was 10.7% in our study, just like wobble; ideally, this value should be as close as possible to zero, which did not occur in any of the study groups, increasingly supporting the idea that a computer system cannot replace the analytical capacity of an anesthesiologist when it is necessary to make major decisions and avoid deviations in anesthetic maintenance of patients.

The strength of this study lies in the use of software developed by the research group, which, despite of using the pharmacokinetic models previously described, is novel given the incorporated adjustments. This is also one of the first studies to incorporate neuromonitoring using the SEDline^TM^ for anesthetic depth, including spectrography to know the point of anesthetic depth in real time as a greater advantage over previous neuromonitoring systems.

Some of the limitations of this study were the number of cases and its descriptive nature that did not allow making comparisons with the system based on monitoring with BIS^TM^ or making statistical inference between the study groups analyzed. In addition, electroencephalography was not performed prior to the study to exclude patients with baseline abnormalities on the EEG, and patients with previous neurological disorders were not considered for exclusion criteria. EEG monitoring was not documented or taken into account as a variable to be measured in the study, only its derived index (PSI^TM^).

## Conclusions

This study, which used a total intravenous anesthesia administration system with propofol and remifentanil guided by a novel neuromonitoring system, the SEDLine^TM^ that provides the PSI^TM^ as an index of anesthetic depth, was used without major complication and appear to be feasible in its use in ASA I and II patients undergoing elective non-cardiac surgeries.

It is necessary to carry out further studies to compare this technology with similar ones and demonstrate their advantages to obtain clinically relevant conclusions and continue optimizing the system. To date, there are no studies that compare closed loop systems with BIS^TM^ vs. closed loop systems with SEDline^TM^; actually, most of them focus on showing the safety of the closed system vs. administration with open system, making necessary, in our opinion, to make progress in establishing which could be the most appropriate anesthetic depth monitor to link and feedback the TCI system.
